# Loss of sensitivity of immunochromatographic test (ICT) for lymphatic filariasis diagnosis in low prevalence settings: consequence in the monitoring and evaluation procedures

**DOI:** 10.1186/s12879-015-1317-x

**Published:** 2015-12-23

**Authors:** Raceline Gounoue-Kamkumo, Hugues C. Nana-Djeunga, Jean Bopda, Julie Akame, Ann Tarini, Joseph Kamgno

**Affiliations:** Centre for Research on Filariasis and other Tropical Diseases (CRFilMT), P.O. Box 5797, Yaounde, Cameroon; Department of Animal Biology and Physiology, Laboratory of Animal Physiology, Faculty of Science, University of Yaounde 1, P.O. Box 812, Yaounde, Cameroon; Department of Animal Biology and Physiology, Parasitology and Ecology Laboratory, Faculty of Science, University of Yaounde 1, P.O. Box 812, Yaounde, Cameroon; Helen Keller International, P.O. Box 14227, Yaounde, Cameroon; Faculty of Medicine and Biomedical Sciences, University of Yaoundé 1, P.O. Box 1364, Yaounde, Cameroon

**Keywords:** Diagnostic tests, Enzyme-linked immunosorbent assay, Immunochromatographic test, Lymphatic filariasis, Sensitivity

## Abstract

**Background:**

Diagnostic tools for lymphatic filariasis (LF) elimination programs are useful in mapping the distribution of the disease, delineating areas where mass drug administrations (MDA) are required, and determining when to stop MDA. The prevalence and burden of LF have been drastically reduced following mass treatments, and the evaluation of the performance of circulating filarial antigen (CFA)-based assays was acknowledged to be of high interest in areas with low residual LF endemicity rates after multiple rounds of MDA. The objective of this study was therefore to evaluate the immunochromatographic test (ICT) sensitivity in low endemicity settings and, specifically, in individuals with low intensity of lymphatic filariasis infection.

**Methods:**

To perform this study, calibrated thick blood smears, ICT and Og4C3 enzyme-linked immunosorbent assay (ELISA) were carried out by night to identify *Wuchereria bancrofti* microfilarial and circulating filarial antigen carriers. A threshold determination assay regarding ICT and ELISA was performed using serial plasma dilutions from individuals with positive microfilarial counts.

**Results:**

All individuals harbouring microfilariae (positive blood films) were detected by ICT and ELISA, but among individuals positive for ELISA, only 35.7 % of them were detected using ICT (Chi square: 4.57; *p*-value = 0.03), indicating a moderate agreement between both tests (kappa statistics = 0.49). Threshold determination analyses showed that ELISA was still positive at the last plasma dilution with negative ICT result.

**Conclusions:**

These findings suggest a loss of sensitivity for ICT in low endemicity settings, especially in people exhibiting low levels of circulating filarial antigen, raising serious concern regarding the monitoring and evaluation procedures in the framework of LF elimination program.

**Electronic supplementary material:**

The online version of this article (doi:10.1186/s12879-015-1317-x) contains supplementary material, which is available to authorized users.

## Background

Lymphatic filariasis (LF), better known as elephantiasis, is a mosquito-borne parasitic disease caused by the nematode parasites *Wuchereria bancrofti*, *Brugia malayi* and *Brugia timori*. This filarial disease is one of the most important public health problems facing many countries in the tropics. It is estimated that 120 million people are infected, and about 40 million disfigured and incapacitated by the disease [1]. In addition to physical disability, LF is also responsible of an important socio-economic burden, with for instance negative impact on marriageability and conjugal life of hydrocele individuals [2]. In 2000, the Global Programme to Eliminate Lymphatic Filariasis (GPELF) was launched by the World Health Organization (WHO) to help eliminating the disease as a public health problem. LF is now among the neglected tropical diseases targeted for elimination by 2020 [3]. To achieve this ambitious goal, the strategy developed by the GPELF is to stop the transmission through large-scale annual treatment (ivermectin or diethylcarbamazine in combination with albendazole) of all eligible individuals living in endemic areas, and alleviate the suffering caused by LF through morbidity management and disability prevention activities [1]. Four programmatic steps - mapping, mass drug administration (MDA), surveillance and verification - were recommended to achieve transmission interruption. In Cameroon, apart from a systematic review reporting quite old LF data [4], very few data were available from limited number of recent field surveys [5, 6]. According to these epidemiological data, either old or recent, the prevalence of LF infection was less than 25 % over the country. In order to follow the GPELF guidelines, a nationwide map of LF was conducted to accurately delineate areas where MDA is required [7]. During the mapping exercise, WHO recommends to classify each surveyed area as endemic or non-endemic, and identify sentinel and spot check sites where baseline data should be collected to provide metrics necessary for the evaluation of the success of the program [8]. A number of tools are currently available for the diagnosis of *W. bancrofti* infection but blood films and ICT were chosen to monitor and evaluate the different programmatic steps necessary to achieve LF elimination [9]. The choice of these diagnostic tools depends upon their sensitivity and specificity, as well as their feasibility in terms of field implementation, technical skills required and cost. Although blood film can overestimate MDA success because of loss of sensitivity when the prevalence and intensity of infection are low, it can be underestimated by ICT since residual antigen levels may persist for up to three years post-treatment in microfilaria-negative individuals [10]. It was shown that ICT card test is very easy to perform, with a high level of sensitivity for the detection of filarial antigen as with ELISA, especially in the laboratory conditions [11, 12]. However, recent evidences indicate that the sensitivity of ICT might be highly variable, depending on age, sex, presence or absence of living adult worms, as well as microfilarial density [13]. Indeed, a positive trend was found between microfilarial density (measure by blood smears) and circulating filarial antigen (CFA), measured either by ICT or ELISA, as well as with the number of adult worms assessed by ultrasound [13, 14]. The objective of the present study was to evaluate, in the field conditions, the ICT sensitivity in low endemicity settings, especially in individuals exhibiting low intensity of LF infection, with the ultimate goal to check whether ICT might remain suitable to evaluate the success of LF elimination program.

## Methods

### Study design and patients

**Fig. 1:**
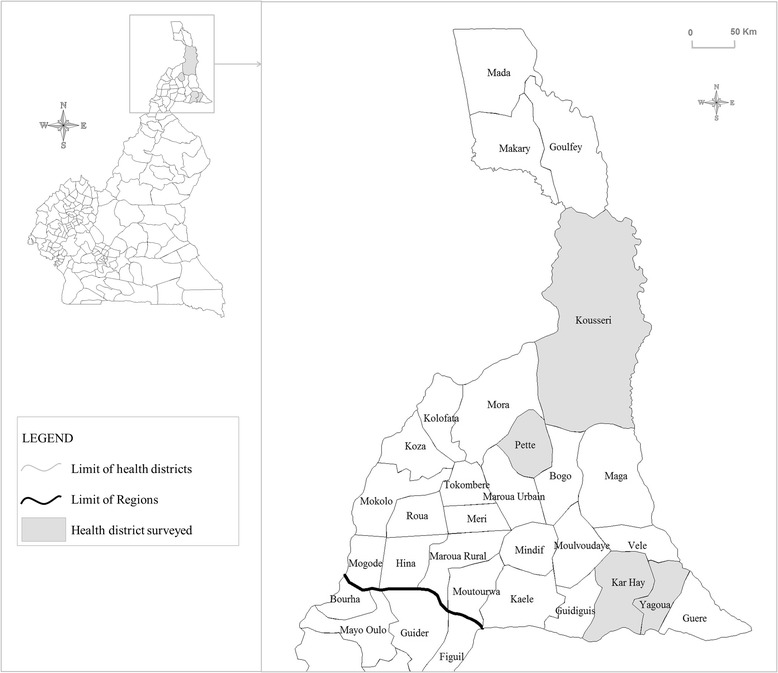
Map showing health districts surveyed in the Far North Region of Cameroon. The grey zones indicate the health districts surveyed

**Fig. 2:**
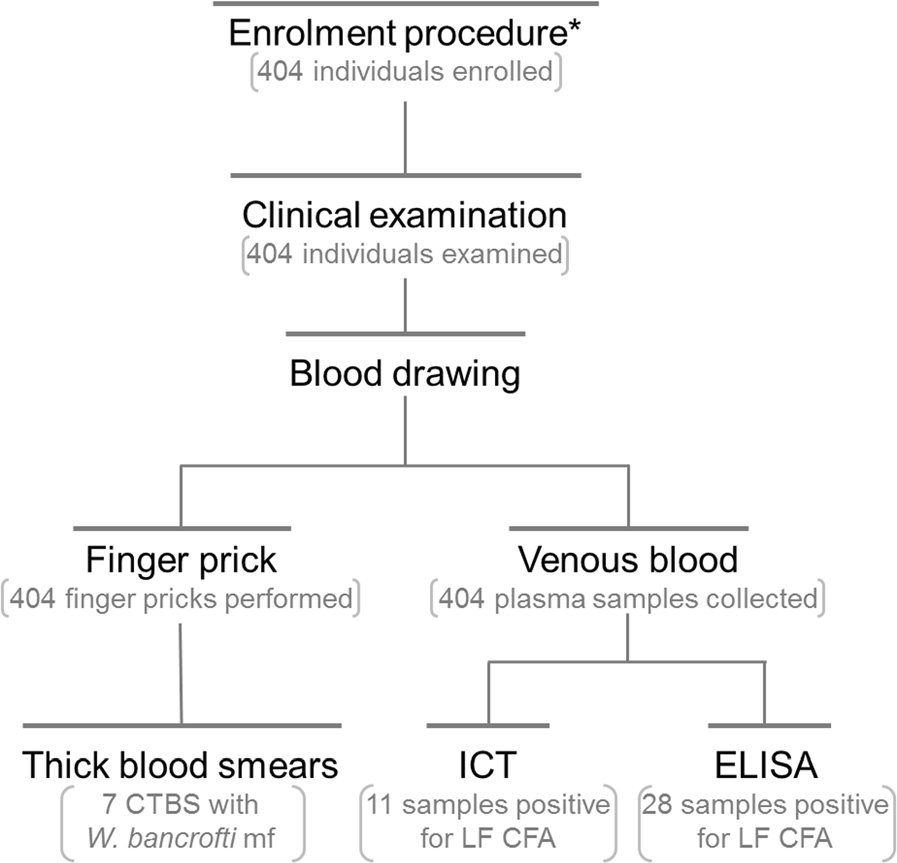
Flow chart detailing the study design and the flow of participants. * All the procedures were performed by night, from 10 PM to 2 AM the next day, on the same individuals. CTBS: calibrated thick blood smear; mf: microfilariae; ICT: immunochromatographic test; ELISA: enzyme-linked immunosorbent assay; LF: lymphatic filariasis; CFA: circulating filarial antigen

The present survey was carried out in two steps following WHO recommendations, to (i) determine where active LF transmission was occurring, and (ii) provide metrics for further evaluation of the success of MDA [8]. To this end, four health districts (Kar-Hay, Kousseri, Pette and Yagoua) located in the Far North Region of Cameroon (Fig. 1) were selected as part of the countrywide mapping operations. The study area delimited by these four health districts was known as an historical focus of LF [4], with prevalence ranging from 0.4 to 22.1 % [15–18], but where no mass antifilarial intervention was ever performed before the outset of our survey. The overall population of these four health districts was 490 470 individuals at the outset of the study. WHO recommends a sentinel site of 300–500 individuals for MDA impact assessments in an implementation unit of 1 000 000 individuals [8]. In each of these health districts (implementation units for health interventions in Cameroon), couples of communities (1–2) with the highest disease or parasite prevalence were chosen as sentinel sites, with the assumption that they were likely to require the longest period of time for interruption of transmission [8]. Fig. 2 displays a chart indicating the different steps of the study including selection and flow of participants as well as sample collection and laboratory processing. In each community, ICT tests were performed to update the prevalence of LF in this area, and night blood smears realized to collect baseline parasitological data to monitor the success of the LF control program [8]. Individuals eligible for this survey were either male or female, aged 5 years old and over. They were invited at a central place in each community (usually at the chief palace) and all volunteers undergo clinical examination for LF clinical signs, and blood sample collection for prevalence and intensity of the infection. The entire procedures were performed by night (from 10 PM to 2 AM the next day) to take into account the nocturnal periodicity of *W. bancrofti* [19].

### Clinical examination

Before undergoing blood drawing and subsequent CFA (ICT and ELISA) and microfilarial (CTBS) detection, a clinical examination was performed to seek for the most striking LF clinical signs. Lower limbs and men scrotum were inspected for elephantiasis and hydrocele, respectively.

### Blood drawing and laboratory processing for circulating filarial antigen (CFA) detection

Five millilitre of venous blood was collected, from each eligible volunteer, in EDTA tube. After centrifugation at 6000 rpm for 5 min, plasma was collected and stored at −20 °C for further serological analyses. CFA detection was performed in the Centre for Research on Filariasis and other Tropical Diseases (CRFilMT) laboratory, using the classical recommended mapping tool (ICT test) to appreciate the prevalence of the infection [8], and the Og4C3 ELISA assay as a confirmation test.

ICT is a rapid card test manufactured by BinaxNow (Scarborough, USA) for the detection of 200 kDa *W. bancrofti* CFA [20]. A volume of 100 μL of each individual plasma sample was added to the sample pad of the card according to the manufacturer protocol. This pad contains a gold-labelled polyclonal anti-filarial antibody that binds to filarial antigen from the plasma. Once the card is closed, the pad comes into contact with a nitrocellulose strip. The antibody-antigen complex then moves along the strip and is trapped by an immobilized anti-filarial monoclonal antibody (AD12.1) present in the strip’s coating. At 10 min, pink control (C) and test (T) lines are visible for all valid positive tests, whereas only the control pink line appears for antigen-negative individuals.

The Og4C3 ELISA test kit, specific for *W. Bancrofti* CFA, was used following the manufacturer (TropBio Pty ldt, Queensland, Australia) recommendations. Briefly, 100 μL of each individual plasma sample, mixed with 300 μL EDTA solution, was boiled at 100 °C for 5 min to release the heat stable CFA in positive specimens. After centrifugation (10,000 rpm for 5 min), 50 μL of supernatant was added, in duplicate, in microtitre plates previously coated with a monoclonal anti-filarial antibody (Og4C3). Serial positive controls from seven standard antigens with known concentrations were added in duplicate in microplate wells, and a negative control also added in duplicate. After overnight incubation, microplates were washed using a wash buffer (provided by the manufacturer); then, 50 μL of rabbit anti-CFA antibody was added in each microplate well, and the latter incubated for one hour at room temperature. Microplates were then washed again and 50 μL of anti-rabbit horse-radish-peroxidase-conjugate added in each well. Finally, microplates were developed by addition of a substrate, and examined on HumaReader HS reader at 405 and 492 nm.

### Calibrated thick blood smears (CTBS) for baseline data collection

Calibrated thick blood smears (CTBS) were performed, following standard protocol developed by the CRFilMT, to assess the intensity of LF infection. Briefly, 50 μL of blood sample was collected from finger-prick, spread on a slide, water de-haemoglobinized and stained with Giemsa. These preparations were examined under a light microscope (magnification x100 or x400). *W. bancrofti* microfilariae were identified and counted, and the results expressed as microfilariae (mf) per millilitre of blood (mf/mL) [21].

### Threshold determination between ICT and ELISA

Plasma samples with positive *W. bancrofti* microfilarial count were tested by ICT and ELISA assays to assess the threshold determination regarding CFA plasma level. Serial plasma dilutions ranging from 1/2 to 1/128 were performed using physiological water (0.9 % NaCl solution) and tested by ICT card. For each sample, the last plasma dilution which yielded negative result to ICT was tested by ELISA to compare threshold determination of these two tests.

### Data analysis

CFA (estimated with ICT and ELISA) and microfilarial (estimated using CTBS) prevalences’ were expressed as the percentage of infected individuals among the total number of individuals examined; the intensity of infection was calculated when the microfilarial count was available as arithmetic means. Sampling fluctuations were estimated either by the 95 % confidence interval (CI) for prevalences - calculated using the Wilson procedure without a correction for continuity [22] - or by the standard deviation (sd) for means. Sensitivity of ICT assays was estimated using ELISA as the reference test. The sensitivity was determined as the percentage of subjects with a positive ICT test among those positive for ELISA; in addition, a Kappa statistics was performed to estimate the strength of agreement between ICT and ELISA assays. The hierarchical weights for agreement associated with kappa statistics was ranged as follows: *k* < 0.00 to indicate no agreement, *k* = 0.00–0.20 to indicate poor agreement, *k* = 0.21–0.40 to indicate fair agreement, *k* = 0.41–0.60 to indicate moderate agreement, *k* = 0.61–0.80 to indicate substantial agreement, and *k* = 0.81–1.00 to indicate almost perfect agreement [23]. All statistical analyses were performed online using VassarStats computational website [24], non-overlapping 95 % CI or *p*-values less than 5 % being considered as statistically significant.

### Ethical statement

The Cameroon National Ethics Committee for Human Research (CNERSH), which is in charge to approve any research involving human subject in Cameroon, is under the authority of the Ministry of Public Health. This study was conducted as part of the action plan of the national program to eliminate lymphatic filariasis in Cameroon, in accordance to the ethical principle for medical research involving human subjects as stated in the Declaration of Helsinki. The survey was approved by, and undertaken under the authority of, the Ministry of Public Health of Cameroon. After approval of the local administrative and traditional authorities, the objectives and schedule of the study were first explained to community leaders and then to all eligible individuals. The latter had the opportunity to ask questions to have full information about their participation in the survey before granting approval. Because of low literacy rate in some areas and the logistics constraints related to the substantial number of individuals to be sampled (this survey was part of the mapping exercise at the national level), a written agreement was not requested. Verbal agreements were recommended by the Ministry of Public Health and obtained from those who agreed to participate, under the discretion of community leaders. Even after the agreement of minors, the approval of their parents or legal guardians was necessary before any procedure. Each team leader was responsible to record agreements and attribute individual code to each participant for anonymous data analysis. At the end of all procedures and examinations, all individuals in each health district found endemic for LF received an anti-filarial treatment (single dose of ivermectin at 150 μg/kg of bodyweight, in combination with albendazole 400 mg) as part of the mass treatment administrated in the framework of the LF elimination program.

## Results

### Prevalence and intensity of LF

Table 1 provides details of the prevalence and intensity of LF infection, using ICT, ELISA and CTBS. In the four health districts included in the present survey, 404 individuals agreed to participate and provided samples for LF diagnosis. All these 404 individuals were tested for ICT, ELISA and CTBS. CFA was detected in 11 (2.7 %; 95 % CI: 1.5–4.8) enrolees using ICT and in 28 (6.9 %; 95 % CI: 4.8–9.8) individuals using ELISA, the difference being significant (Chi square: 7.79; *p*-value = 0.0053). CTBS revealed that 7 (1.7 %; 95 % CI: 0.8–3.5) of these 404 individuals harboured mf, with an intensity of infection of 7.49 mf/mL (sd: 70.77).

**Table 1:** Prevalence and intensity of LF infection in four health districts of the Far North Region of Cameroon

Implementation unit	Number examined	% prevalence of ICT (95 % CI)	% prevalence of ELISA (95 % CI)	% prevalence of CTBS (95 % CI)	Mean mf count^a^ (sd)
Yagoua	144	6.3 (3.3–11.5)	9.7 (5.9–15.7)	4.2 (1.9–8.8)	19.72 (116.86)
Kar-Hay	131	1.5 (0.4–5.4)	9.9 (5.9–16.2)	0.8 (0.1–4.2)	1.37 (15.73)
Pette	87	0.0 (0.0–4.2)	1.1 (0.2–6.2)	0.0 (0.0–4.2)	0.00 (0.00)
Kousseri	42	0.0 (0.0–8.4)	0.0 (0.0–8.4)	0.0 (0.0–8.4)	0.00 (0.00)
Overall	404	2.7 (1.5–4.8)	6.9 (4.8–9.8)	1.7 (0.8–3.5)	7.48 (70.77)

^a^mf count is expressed as the number of mf per mL of blood (mf/mL)

### Agreement between diagnostic tests and threshold determination of LF CFA

All individuals with mf were identified using ICT and ELISA, but among individuals positive for ELISA, only 10 (35 %) were detected using ICT (Chi square: 4.57; *p*-value = 0.03), the value of the kappa statistics (*k* = 0.49) indicating a moderate agreement between ELISA and ICT [see Additional file 1: Dataset S1]. Threshold determination analyses showed that ICT was positive at some dilution factors for different plasma samples, but ELISA was still positive at the last plasma dilution for which ICT yielded a negative result.

### Relationship between LF macroscopic lesions and CFA

Among the 404 individuals examined, none presented with elephantiasis, whereas 21 (5.2 %; 95 % CI: 3.4–7.8) were suffering from hydrocele (8 in Yagoua [5.6 %; 95 % CI: 2.8–10.6], 12 in Kar-Hay [9.2 %; 95 % CI: 5.3–15.3] and 1 in Pette [1.1 %; 95 % CI: 0.2–6.2]). *W. bancrofti* circulating antigen from 20 of these individuals suffering from hydrocele were identified by ELISA whereas only two of them were detected using ICT [see Additional file 1: Dataset S1].

## Discussion

Diagnostic tools are useful for (1) mapping the distribution of LF to delineate areas where MDA are required, (2) assessing the impact of MDA to determine when they can be stopped after transmission has been interrupted, and (3) ensuring strong surveillance procedures post–MDA to monitor possible resurgence [8, 12]. Although some of these tools are difficult to implement in field conditions, others lack sensitivity, especially when the intensity of infection is low. The present study showed low level of LF infection in the study area. A moderate agreement (kappa statistics = 0.49) was found between ICT and ELISA, the threshold determination analyses showing that ELISA was still positive at the last plasma dilution with negative ICT result.

ICT has been chosen by the GPELF since more than 10 years to serve as the reference test in the framework of LF global elimination, and has almost always been presented to be as sensitive as ELISA [12, 20]. However, we surprisingly found in the present study that ELISA was significantly more sensitive than ICT, with a moderate - at the edge of a fair - agreement for the detection of LF circulating antigen. It was previously demonstrated that amicrofilaremic adult worm carriers or persons with ultra-low mf density could be at increased risk for false-negative ICT results [11, 25], the sensitivity of Og4C3 ELISA being reduced at about 75 % in individuals harbouring ≤50mf/mL [14]. Indeed, the mean microfilarial density found in the present study was equal to 7.49 mf/mL (sd: 70.77), and serial dilutions of mf positive samples confirm the ICT loss of sensitivity when the CFA concentration was diluted and consequently drops down as observed elsewhere [20, 25]. A high correlation was previously reported between mf densities and ELISA titres, ICT results as well as the numbers of adult worms, the degree of relationship being improved with increasing mf densities [13]. This can suggest that the mf density can be used as a proxy to estimate the adult worm carriage, and therefore the CFA level in the plasma. Given the low sample size of mf infected individuals in our study (only 7 found), we didn’t attempt to correlate the mf densities to the ICT outcome or ELISA titres. One must however pointed out that ELISA was always positive when ICT yielded a negative result.

The loss of ICT sensitivity in people harbouring low mf densities or low CFA level might be a limiting factor for the use of ICT in low prevalence settings where the likelihood to find individuals with low intensity of infection is high. In addition, since repeated treatments lower individual mf density, ICT might not be appropriate for the monitoring of the impact of MDA on the endemicity or transmission of the infection. These results corroborate those obtained during the validation of a newly developed test (Alere Filariasis test strip) in Liberia where mass treatments were distributed just six months before the field study [26]. Indeed, the Alere Filariasis test strip was significantly more sensitive than the ICT card test, by detecting 26.5 % more individuals with CFA. Also, in a prevalence study from a low endemic area in Bangladesh, the sensitivity of ELISA was shown to be significantly higher than that of ICT after five rounds of MDA [25]. More importantly, ICT has misclassified this area as no longer requiring treatments and ELISA therefore proposed for post-MDA surveillance. However, Og4C3 ELISA can also experience loss of sensitivity (even at a reduced magnitude as compared to ICT), and some limitations such as the necessity of laboratory infrastructure for performance and interpretation of the results [20]. Therefore, the development and validation of improved and practical methods for assessing filariasis transmission as well as post-MDA surveillance improved protocols for early detection of persistent or resurgent LF transmission are still needed [9].

### Limitations of the study and possible bias

The present study was part of the Cameroon wide LF mapping exercise, following the WHO recommendations. As such, it was not designed to compare ICT and ELISA assays. Although sample size was sufficient to draw solid enough conclusion regarding antigenaemia detection using ICT and ELISA (the power of statistical tests were ~80 %), we didn’t attempt any inferential statistics for threshold determination analysis. Indeed, as a consequence of the low prevalence and intensity of LF in the study area, a reduced number (seven) of subjects was mf positive and their sera available for the threshold determination analysis. However, the trend observed was very interesting and might deserve further investigations. Also, at the contrary of ELISA, ICT was not designed to be tested on diluted samples since this could affect the performance of the test in unknown ways. In the present study, these dilutions were performed to mimic a reduced concentration of filarial antigen in the plasma, and the ICT test remained positive at some dilution factors. The test line was clearly visible at 10 min for each plasma dilution positive for ICT, whereas negative dilutions displayed no test line, even beyond the 10 min recommended by the manufacturer. We hypothesised that such dilution might reduce the affinity of the antigen-antibody complex and therefore the sensitivity of the test. Similar trends were observed during the analytical comparison of the sensitivity of ICT and its next generation homologue (Alere Filariasis test strip). Indeed, parallel testing performed from serial dilutions of a filarial antigen (DATH) [26, 27] have shown that the ICT card test was weakly positive with antigen diluted 1/4, higher dilutions being clearly negative, and the minimum concentration detected by the test strip was two to four fold lower than the minimum detected using ICT card test [26].

## Conclusion

The elimination of LF is now on top of agenda of control programs and stakeholders; our findings suggest that care should be taken when using ICT card test for monitoring and evaluation procedures, and development of highly sensitive tests are of interest. Indeed, very sensitive polymerase chain reaction (PCR) assay have been developed, some being able to detect an amount of DNA equivalent to half mf per ml of blood [28]. However, such assays are highly labour-intensive and expensive for routine use in endemic areas [20]. We welcome the next generation of ICT card test, the Alere Filariasis test strip, which has been developed and tested both in laboratory and in the field condition in Liberia. Although the newly developed test strip appears to be more sensitive, affordable and most suitable for routine field assays, it is based on the same principle as with the ICT card test and reagents used are the same [25]. Therefore, more field testing might be of importance to validate this new test, especially in settings with low prevalence and intensity of infection where loss of sensitivity with ICT card tests has been observed. This is more than ever important since this test will likely replace the ICT card test and used to assess the success of MDA on the transmission of LF, and therefore guide the decision to stop MDA.

## Data availability statement

The dataset supporting the results of this article is included within the article and its additional file.

## Additional file

Additional file 1: Dataset S1. Database for lymphatic filariasis survey in four health districts in the Far North Region of Cameroon (XLS). NA: non applicable; 0: negative; 1: positive. (XLS 92 kb)
